# TME16/459: S.I.TE. Project: Spinal injury and Telemedicine

**DOI:** 10.2196/jmir.1.suppl1.e123

**Published:** 1999-09-19

**Authors:** S Boccaccio, P Marano, R Saracco, N Dal Degan

**Affiliations:** ^1^Istituto Ortopedico Villa Salus Strada Provinciale Brucoli AugustaAugustaItaly

**Keywords:** Internet, Spinal Cord Injury, Telemedicine

## Abstract

**Introduction:**

A meeting held in Augusta, in November '98, proposed the launch of a Telemedicine application in order to improve spinal trauma rehabilitation protocols. This medical area, as many others, is still populated by diverse ideas and doubts on the possible protocol of intervention: a common protocol tying together surgical, rehabilitation, orthopaedic and neurological medicine can lead to significant results.

**Methods:**

Scope of this project, is to realise a network (virtual-line) using Internet, connecting Italian and German spinal centres interested on this protocol, and to allow for the exchange of data, clinical reports, images, with different levels of interaction:

1. Store and forward
  - E. mail;
  - Attached documents;
  - News group
2. Real time
  - Videoconferencing system and electronic board.

External users may have restricted access to specific information, although we can see the results of the work in a web site always kept updated

**Results:**

The specification phase is now complete and at present the work is progressing towards the realisation phase.

**Discussion:**

This application is very interesting from different points of view:

1. It is a new Telemedicine application.
2. It uses Internet, hence transportation cost are marginal and its reliance on de facto standard browser eases further applications development and portability.
3. It fosters a new approach to co-operative research in medicine.

